# Tackling SNR at low-field: a review of hardware approaches for point-of-care systems

**DOI:** 10.1007/s10334-023-01100-3

**Published:** 2023-05-18

**Authors:** Andrew Webb, Thomas O’Reilly

**Affiliations:** Department of Radiology, C.J. Gorter MRI Center, Leiden University Medical Center, 2333 ZA Leiden, The Netherlands

**Keywords:** Low-field MRI, Magnets, RF coils, Point-of-care, Grounding, Shielding, Litz wire

## Abstract

**Objective:**

To review the major hardware components of low-field point-of-care MRI systems which affect the overall sensitivity.

**Methods:**

Designs for the following components are reviewed and analyzed: magnet, RF coils, transmit/receive switches, preamplifiers, data acquisition system, and methods for grounding and mitigating electromagnetic interference.

**Results:**

High homogeneity magnets can be produced in a variety of different designs including C- and H-shaped as well as Halbach arrays. Using Litz wire for RF coil designs enables unloaded *Q* values of ~ 400 to be reached, with body loss representing about 35% of the total system resistance. There are a number of different schemes to tackle issues arising from the low coil bandwidth with respect to the imaging bandwidth. Finally, the effects of good RF shielding, proper electrical grounding, and effective electromagnetic interference reduction can lead to substantial increases in image signal-to-noise ratio.

**Discussion:**

There are many different magnet and RF coil designs in the literature, and to enable meaningful comparisons and optimizations to be performed it would be very helpful to determine a standardized set of sensitivity measures, irrespective of design.

## Introduction

The recent growing interest in mid-field (0.1–1 T), low-field (0.01–0.1 T) and point-of-care (POC) MRI has been driven by the concept of increasing the accessibility of a traditionally extremely expensive imaging modality. The increased accessibility of mid-field systems can be related primarily to reduced purchase, maintenance and infrastructural costs, and increased availability for patients with contraindications such as medical implants, compared to conventional (1.5–3 T) field strengths. POC low-field systems, which are designed to be portable, increasing access to MRI by enabling it to be used in situations in which it has not previously been possible: examples include the intensive care unit, emergency rooms and ambulances in the developed world, and sites in lower and middle income countries (LMICs) which lack the finance and/or infrastructure for conventional MRI systems.

The major technical challenge of mid-field and low-field MRI in general is, as expected, that of signal-to-noise ratio (SNR). For POC systems, the limited *B*_0_ field homogeneity is a challenge, arguably equal to that of low SNR. This is primarily due to the reduced size of such systems, which means that the imaging field-of-view lies very close to the inner surface of the magnet. From a hardware point-of-view, the major contributing components to SNR are the magnet, in terms of both strength and homogeneity, the RF coil(s) in terms of sensitivity, and the receiver chain in terms of total noise figure. Each of these is considered in this review article.

Starting from well-known equations, the magnetization (*M*_0_) per unit volume of tissue can be expressed as:

1 $$M_{0} = \frac{{N\gamma^{2} h^{2} I\left( {I + 1} \right)B_{0} }}{{12\pi^{2} k_{{\text{B}}} T_{{\text{s}}} }}$$

where *N* is the number of spins per unit volume, *γ* is the gyromagnetic ratio, *h* is Plank’s constant, *I* is the spin angular momentum, *B*_0_ the main magnetic field, *k*_B_ Boltzmann’s constant, and *T*_s_ the sample temperature, which is assumed to be body temperature. The magnitude of the MR signal (*S*) in the rotating frame (ignoring relaxation) is given by:

2 $$S = \frac{{K\omega_{0} B_{1}^{ - } M_{0} V_{{\text{s}}} }}{i}$$

where *i* is the current in the coil, and assuming that a single coil is used for transmit and receive the conventional *B*_1_^+^/*i* term can be replaced by *B*_1_^−^/*i*, *K* is a numerical factor (between 0 and 1) which essentially is a measure of the integrated sensitivity of the coil compared to that if the *B*_1_ field were perfectly uniform, *V*_s_ is the volume of the sample, and *ω*_0_ is the resonance frequency.

The noise (from both the RF coil and the sample) is given by:

3 $$N_{{\text{s}}} = \sqrt {4k_{{\text{B}}} TR\Delta f}$$

where *N*_s_ is the root mean square noise voltage, *R* the total system resistance and Δ*f* the measurement bandwidth. There are two components to the total resistance, body (sample) loss and RF coil loss, which can be represented as respective resistances, *R*_body_ and *R*_coil_: the total resistance is simply the summation of these two quantities. Body loss can be further subdivided into magnetic losses due to *B*_1_-induced currents in the body and electric losses due to capacitive coupling between the coil and the body [1–6]. In general, it is assumed that if the RF coils are properly constructed, then the body loss is dominant at clinical field strengths, unless very small receiver coils are used. For low-field systems, coil noise represents a substantial component of the total system noise [5–7].

In terms of mid-field MRI, there have been many reports recently associated with systems operating at 0.55 T [8–19]. Somewhat naively, one might expect the SNR to be approximately one-quarter that at 1.5 T. However, a number of factors work to the advantage of the lower field, and reduce the loss factor. Tissue *T*_1_ relaxation times are shorter at 0.55 T, with the *T*_2_ values being roughly equal, and the *T*_2_* time longer, particularly in tissues where there is a strong contribution from tissue micro-inhomogeneities or strong dephasing due to the presence of iron (e.g., liver) or air (e.g., the lungs). For bSSFP sequences the signal intensity is proportional to *T*_2_/*T*_1_, and so are more SNR efficient at lower fields. In addition, the acquisition bandwidth can be reduced by a factor-of-three compared to that at 1.5 T (assuming that one maintains the water/fat shift constant in terms of the number of pixels), reducing the Johnson noise. Marques et al. [20] provided a theoretical analysis of the optimization of sequence parameters as a function of field strength, and concluded that the *B*_0_ dependence was approximately linear, rather than a 3/2 power relationship. Campbell-Washington et al. have provided experimental demonstrations at 0.55 T that the use of highly efficient sequences such as long spiral readouts, or turbo spin echo with full 180° refocusing pulses or balanced steady state free precession sequences with higher tip angles than used at 1.5 T [8–19], can increase the SNR at lower fields, with the extreme case of lung imaging actually giving higher SNR at 0.55 T than at 1.5 T. For most tissues, it has been estimated [21] that the SNR at 0.55 T is approximately 70% that at 1.5 T. The same form of analysis can be performed even for whole-body ultra low field systems (< 0.01 T), e.g., the work initially presented at 6.5 mT by Sarracanie et al. [22], based on a previous electromagnet [23, 24], and follow-up publications [25–27], in which the very homogeneous *B*_0_ field enabled efficient balanced SSFP sequences to be run, with an impressive resulting SNR given the very low *B*_0_ field.

For small, portable POC systems based on permanent magnets, there are many more design parameters that need to be considered to optimize SNR, and there are strong interdependencies between these parameters. In this regard, the critical differences between a POC and conventional MRI system include that for a POC system the *B*_0_ field is much more inhomogeneous, the gradient strengths are much weaker, the available space is much less and so the physical distance between RF coil, RF shield and gradient coils is very small. Some of the issues mentioned in the discussion of the 0.55 T system are also relevant, in particular the lower *T*_1_ tissue value, which increases the available SNR per unit time. However, unlike for a whole-body 0.55 T system, in general the acquisition bandwidth cannot be significantly decreased compared to a clinical scanner due to the relatively inhomogeneous *B*_0_ and the limited gradient strength, otherwise significant point-spread-function (PSF) broadening/imaging distortions would occur in the frequency encoding process (phase encoding is essentially immune to this effect). The inhomogeneous *B*_0_ also means that balanced SSFP, echo planar imaging (EPI) and spiral readouts are challenging to implement.

An example of the design trade-offs is that increasing the diameter of the magnet has the disadvantage of reducing the *B*_0_ field, but the advantage of the larger bore allowing the RF shield to be located further away from the RF coil, increasing the coil sensivity. For this larger diameter magnet, the gradient coil diameter can also be increased which reduces the gradient efficiency but increases the usable imaging volume. A larger magnet also reduces the *B*_0_ inhomogeneity over a given field-of-view and so the acquisition bandwidth can be decreased. As a result of some of these design trade-offs, there are a wide variety of designs and design philosophies of magnet, gradient coils, and RF coils for POC systems [22, 28–37]. The final SNR is, therefore, a complicated function of all of these parameters, which constitute the main body of this review article.

## System design for POC systems

(i) **Magnet**

There are two basic designs currently used for POC low field MRI systems: C- and H-shaped yoked magnets, and Halbach-based arrays. There are relative advantages and disadvantages of each of the designs. A C- or H-based system is much easier to construct. In addition, it is a more open structure. However, the Halbach system is much safer to construct due to the magnetic forces being divided over thousands of small magnets. Gradient coils for the parallel plates are planar, which are intrinsically less efficient than the cylindrical ones which used in a Halbach array. Radiofrequency (RF) coil design is similar, although coupling to the gradient coils is higher in the Halbach design. As outlined previously, the key elements in terms of sensitivity are the *B*_0_ field strength and the homogeneity.

The basic design for C- and H-shaped magnets are two pole pieces of magnetic material such as neodynium boron iron or samarium cobalt, each disc shaped and of equal strength and situated above and below the imaging field of view. Normally, each disc is identical in construction, with the material polarized in the vertical direction. The discs are placed with the same polarity in the vertical direction so that the magnetic field add together in the imaging volume. The two discs are connected by a ferromagnetic yoke (usual made of steel), which is not magnetically saturated, but which increases the magnetic field strength between the two discs. There is an optimum spacing between the discs, in relation to the radius of each disc, which maximizes the B0 field homogeneity [38, 39]. To further improve the homogeneity, smaller permanent magnets can be placed in suitable locations and/or electrical systems for shimming incorporatedfor low-field MRI applications [34, 35, 40, 41].

Enhancing the magnetic field on one side of a magnet array while cancelling the field on the other side was first demonstrated by Mallinson [42]. Halbach [43, 44] laid down a set of rotation rules which gave rise to different multipole field modes inside a cylindrical magnet array: the magnetization along the periphery of the circular cross-section at an azimuthal angle *θ* should be arranged at an angle *α* = (*N* + 1)*θ*, where *N* defines the mode. For *N* = 1, there is a 2*θ* variation which produces a dipole field structure, *N* = 2 gives a quadrupole field and so on. The Halbach configuration has been shown to produce the strongest possible field per unit mass of magnetic material [45, 46]. The rotation rules given by Halbach work best for infinitely long cylindrical arrays with a circular cross-section: in the dipole configuration one obtains a perfectly homogeneous field distribution at the centre of the array. However, practical Halbach cylindrical magnets of finite length suffer from significantly lower field homogeneity. Much research has concentrated on improving the field homogeneity of a finite-length circular cross-section magnet array consisting of a large number of discrete magnet elements. Parameters which have been optimized include the rotation angle of individual magnets, spacing between separate rings of magnets, and the strength and orientation of the magnets themselves [29, 47–58].

**Fig. 1:**
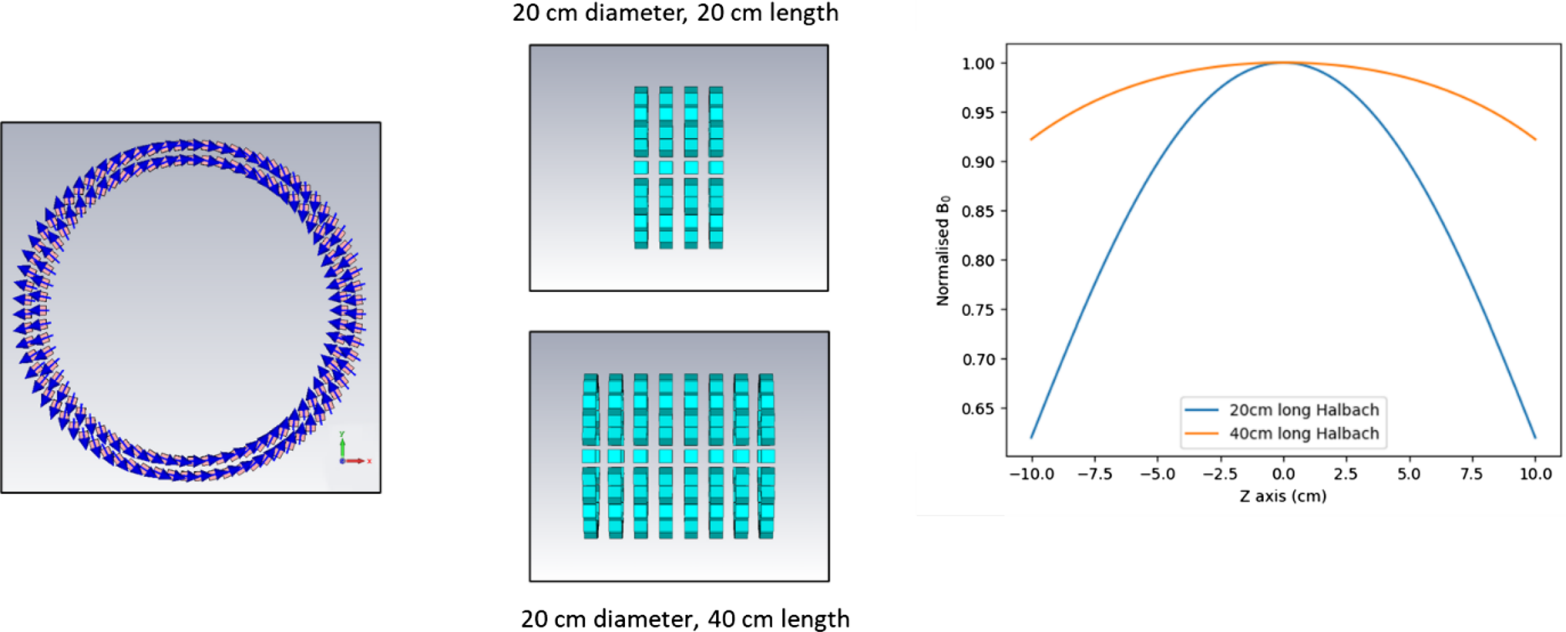
Schematics of a Halbach array constructed from a large number of individual magnets. (left) Front view of the orientation of the individual magnet dipoles (north/south). Centre and right show that a larger length:diameter ratio produces a more homogeneous *B*_0_ field (the line is plotted along the centre of the magnet in the front-to-back direction)

The magnetic flux density (*B*) in an ideal Halbach dipole array is given by:

4 $$B = B_{{\text{R}}} \ln \frac{{r_{{\text{o}}} }}{{r_{{\text{i}}} }}$$

where *B*_R_ is the remanence of the magnetic material, and *r*_o_ and *r*_i_ the outer and inner radii of the annular magnet. In addition, since continuous pieces of magnetic material with spatially varying magnetization cannot be produced in practice, the arrays consist of a number of discrete individual magnet elements (these were termed NMR-Mandhalas, but for consistency with existing literature we use the term Halbach to include the discrete Mandhala designs). These structures generate a smaller field than would originate from a continuous magnetic material, because the space between *r*_o_ and *r*_i_ is not completely filled with magnetic material. There is a reduction of about 50% in *B*_0_ for magnets with a square cross-section, and ~ 25% flux reduction magnets with octagonal or circular cross-section. Overall, discrete Mandhalas and continuous Halbachs produce almost identical *B*_0_ field per unit mass of magnetic material. In terms of B_0_ homogeneity, discretization using polygonal magnets increases the inhomogeneity by ~ 20–30% compared to a continuous Halbach, with the square/cubic magnets producing up to an order of magnitude poorer homogeneity. Blumler [44, 45] also reported that these values depend on the number of magnets used in the discretization, and almost exponentially on the radius of the volume over which the homogeneity is determined. Figure 1 shows schematics of a discrete Halbach magnet, as well as an illustration of the dependence of the *B*_0_-homogeneity on the length:diameter ratio.

Whether the designed system is C-/H-shaped or a Halbach array, it requires substantial *B*_0_ shimming to reach its theoretical optimal homogeneity. This is primarily due to small errors in exact magnet positioning, and small non-uniformities in the properties of the magnetic materials. The major challenge is that, since the imaging field-of-view is a substantial fraction of the total magnet volume, the inhomogeneities are large compared to conventional clinical MR magnets, and also contain higher order terms close to the magnet surfaces. *B*_0_ shimming can be performed using small permanent magnets (fixed shimming) or by passing current through extra conductive shim coils (case-by-case shimming), as well as a combination of both approaches. Typically, *B*_0_ homogeneity is measured using a three-axis Hall probe with some form of robotic positioning device. Uberruck et al. [59] investigated two different approaches for using permanent magnets to shim C- and H-shaped magnets for a table-top system, one which used a hexagonal array of 37 small magnets in a hexagonal arrangement, and the other which used four rings of permanent magnets which could be physically displaced. Lopez et al. [60] presented a passive shim design method based on expressing the shim distribution as a sum of orthogonal functions, and solving for the coefficients to determine the positioning of sets of permanent magnets. McDowell and Conradi [61] designed a set of very thin wire higher order electrical shim coils for a small tabletop system based on a dipolar magnet. Terada et al. [40] used a combination of permanent magnets and a single electrical shim coil for a compact 0.3 T system for imaging the human hand. For Halbach arrays, Parker et al. [62] described a system based on a core unit of two nested Mandhalas comprising hexagonal magnets, and a shim unit consisting of identical hexagonal magnets in outer shells. Wenzel et al. [63] used an iterative scheme and genetic algorithm to design as set of shim magnets for a small Halbach magnet with an imaging field-of-view of 4 cm. Wang et al. [64] proposed a shimming method based on sheets of permanent magnet material targeting at spherical harmonic basis up to the 3rd order including dedicated composition of *Y*(4*Z*^2^−*X*^2^−*Y*^2^), *Z*^3^ and *X*(4*Z*^2^−*X*^2^−*Y*^2^) (*n* = 3, *m* = − 1, 0, 1) with cross terms to implement structural field compensation.

(b) ***RF coils***

The basic aim of RF coil design, in terms of maximizing SNR, is to ensure that the coil loss represents as small a fraction of the body loss as possible. This is not difficult at the frequencies used for conventional clinical MRI, but becomes more challenging at low frequencies, and indeed is not attainable at frequencies in the tens to hundreds of kHz range [65].

- *Geometry*

RF coil designs can be both transmit and receive (Tx/Rx), or have separate coils for transmit (Tx) and receive (Rx), which must obviously be isolated from one another, using either geometrically orthogonal designs or switchable detuning circuits. The transverse orientation of the B_0_ field in most POC systems (C-shaped, H-shaped and Halbach, although not O-shaped [37]), allows the use of a solenoidal geometry for the RF coil. Variations of the solenoid, which more closely conform to the geometry of the head, are also commonly used. As shown many years ago, the solenoid is between a factor-of-two and three more sensitive (*B*_1_/*I*) [66] than a linear saddle or birdcage coil (of course a quadrature birdcage or saddle coil cannot be used in this configuration), with a slightly poorer *B*_1_ homogeneity measured over the active region of the coil. If separate Tx and Rx coils are used, then a solenoid is used as the receive element and either a second solenoid (the two solenoids must each contain actively switchable detuning circuits) or an intrinsically-geometrically-decoupled saddle coil can be used as the transmit element.

It is worth noting that some systems, both academic [67–69] and also the Hyperfine Swoop, have incorporated arrays of receive elements, similar to the situation for clinical MRIs. These receive elements are loop coils with various geometries and sizes. To date, no comparison has been made between the SNR of these arrays compared to a single receiver, and there is little information on parallel imaging performance. Although the geometry (g-factors are likely to be relatively large given the low influence of the sample on coil isolation, there may be cases where the SNR is sufficiently high such that meaningful reductions in total scan time can be achieved without losing significant diagnostic utility.

(b) *Conductor dimensions*

As covered in many publications [70], general fabrication rules for RF coils include the recommendation that the total length of the conductor should be less than *λ*/20 such that substantial wavelength effects are not present. Capacitive segmentation is standardly used in high frequency RF coils as a method to reduce dielectric losses in the body, phase shifts along the length of the coil conductor, and SAR arising from conservative electric fields around individual capacitors in the impedance matching network. The use of capacitive segmentation has been discussed much less for low field coils, but nevertheless is worth incorporating. For example, the wavelength at 2 MHz is ~ 150 m in a copper conductor, although the effective wavelength may be shorter in helices due to compression effects (inter-turn capacitance between turns). This corresponds to a *λ*/20 of 7.5 m, which for a circular solenoid of diameter of 27 cm, represents only ~ 9 turns.

**Fig. 2:**
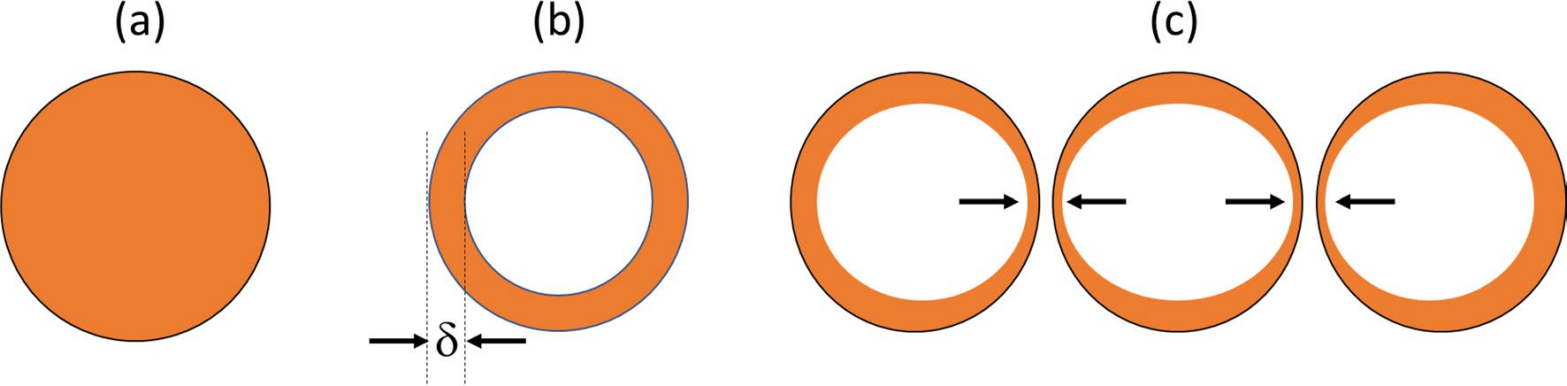
Schematic of the current distribution in a circular cross-section conductor. **a** At zero-frequency direct current (DC) the current is distributed uniformly throughout the wire. **b** Illustration of the skin effect for an AC current, in which the current is maximum at the outside of the conductor, and falls off exponentially with depth with the exponent given by the skin depth (*δ*). The decrease in effective cross-sectional area means that the AC resistance is higher than the DC resistance. **c** Illustration of the proximity effect (shown by the arrows) for a number of parallel conductors. This reduces even further the cross-sectional area through which the current flows, increasing its resistance

In terms of how large the diameter of the conductor should be, and how close the turns should be, there are two effects which need to be considered, shown schematically in Fig. 2.

The resistance of a solenoidal coil, corresponding to Johnson noise, is given by [2]:

5 $$R_{{{\text{coil}}}} \cong \frac{{3\zeta n^{2} a}}{l\sigma \delta }$$

where *ζ* is the proximity effect factor (see below), *σ* is the conductivity of the conductor, n is the number of turns, *a* is the coil radius and l the coil length, and *δ* is the frequency-dependent skin-depth, in turn given by:

6 $$\delta = \sqrt {\frac{\rho }{{\pi f\mu_{0} }}}$$

where *ρ* is the resistivity, *f* the frequency, and *μ*_0_ the magnetic permeability. The effective cross-sectional area, *A*_eff_, which carries current is given by:

7 $$A_{{{\text{eff}}}} = \pi d_{0} \sqrt {\frac{\rho }{{\pi f\mu_{0} }}}$$

The DC resistance of a wire conductor is inversely proportional to the square of the wire diameter, whereas the skin effect is linearly proportional to the diameter, so overall the ratio *R*_AC_/*R*_DC_ decreases inversely with wire diameter. The skin depth at 2 MHz in copper is ~ 47 μm, meaning that 95% of the current is within the outer 0.15 mm. So for example, the AC resistance is ~ 10 times the DC resistance for a 2 mm diameter copper wire.

If two wires carrying AC current in the same direction (i.e., parallel wires) are brought into close proximity then the spatial distribution of the currents within each wire changes. The magnetic field of the first wire induces eddy currents in the second wire, that form loops along the wire. These eddy currents flow in the same direction as the current on the side of the second wire facing away from the first wire, and back in the opposite direction on the side of the second wire facing the first wire. In other words, the overall effect is to redistribute the current in the second wire into a thin cross-sectional area on the side furthest away from the first wire. Since the current is concentrated into a smaller area of the wire, the resistance is increased: this is known as the proximity effect. This effect is shown in Fig. 1c. If the wires in the RF coil are wound very close together then the proximity factor can be very large, up to a factor of 10 if large conductors are used. However, if a separation between the wire centres of twice the wire diameter is used, then the proximity effect drops to ~ 10–15% [66].

Based on these results, the optimum wire diameter for a solenoid would be several cm (see for example, Fig. 4.7 in Chen and Hoult [70]). However, given space restrictions within a typical POC system this diameter is simply not practical, and so rather than use suboptimal thin single conductors, the solution is to use Litz wire. This consists of many thousands of very thin insulated strands of wire, each one which has a diameter smaller than the skin depth, which are braided around each other to ensure that equal current flows in each strand. For Litz wire, containing *N*_s_ strands with diameter *d*_litz_, if *d*_litz_ <  < *δ*, then the proximity effect can be estimate from:

8 $$A_{{{\text{eff}}}} = N_{{\text{s}}} \frac{{\pi d_{{{\text{litz}}}} \delta }}{4}$$

Hoult showed that the *Q*-factor is optimized using a wire separation of approximately 1.5 times the wire radius [66], but for Litz wires the turns can be placed very close together without any proximity effects. For frequencies between 1.4 and 2.8 MHz a wire diameter of ~ 30 microns is optimal. Litz wire has shown to be a feasible approach for constructing RF coils up to 10 MHz [71].

One of the most detailed studies involving Litz wire was by Resmer et al. [72] who derived parameters for optimizing the *Q*-factor and SNR for a multi-turn surface coil at 0.01 T. For solid wire, at low frequencies (< 1 MHz) the skin-effect is much more pronounced than the proximity effect, and so *Q* values increase as the square root of the frequency. As the frequency approaches the self-resonance frequency of the coil, then dielectric losses arising from the self-capacitance begin to play a larger role, and these increase as the third power of frequency. For Litz wire, at low frequencies DC losses are greater than the skin depth and so the *Q* value increases linearly with frequency. At higher frequencies the proximity effects become higher, and beyond its maximum value the *Q* is proportional to 1/*f*. Including dielectric losses reduces the maximum *Q* value, and also shifts it to lower frequencies.

In addition to coil losses there are three other loss mechanisms: inductive and dielectric losses which are both associated with the sample, and radiation losses. From Maxwell’s equations, the alternating *B*_1_^+^ field induces magnetically induced (inductive) losses, also known as eddy current losses, in a conducting sample. These losses can be represented as an effective resistance *R*_m_ in series with the receiving coil, using a model originally suggested by Gadian and Robinson [5]. The value of *R*_m_ is proportional to the square of the operating frequency and the sample conductivity. Dielectric losses result from electrical lines of force, associated with the distributed capacitance of the RF coil, passing through the sample. Gadian and Robinson [5] showed that these dielectric losses could be modelled as a parallel stray capacitance between the coil and the sample, with the conducting sample represented as a parallel resistor (*R*_e_) and capacitor. At low frequencies *R*_e_ is proportional to the fourth power of frequency, in the high frequency limit to the square of the frequency. The higher the sample conductivity the higher the value of *R*_e_ and the higher the losses. *R*_e_ is also proportional to the square of the inductance of the coil. Radiation loss, which represents energy radiated away from the coil rather than into the sample. Radiation loss can be correlated to an equivalent resistance, *R*_radiation_, which is proportional to the fourth power of both frequency and coil radius. Except for very large coils at very high frequencies, the coil resistance is much higher than the radiation resistance.

Experimentally one can measure the *Q* value as a function of frequency to determine the transition between coil noise dominance and body noise dominance [65]. Experimentally this is best performed using two pick up loops placed either side of the coil, and measuring the S_21_ scattering parameter [73]: the *Q* value can then be estimated as the full-width-half-maximum (FWHM) of the peak. By defining a loading factor (LF):

9 $${\text{LF}} = 1 - \frac{{Q_{{{\text{loaded}}}} }}{{Q_{{{\text{unloaded}}}} }}$$

If the LF > 0.5 then coil noise dominates, and if LF > 0.5 coil noise dominates.

**Fig. 3:**
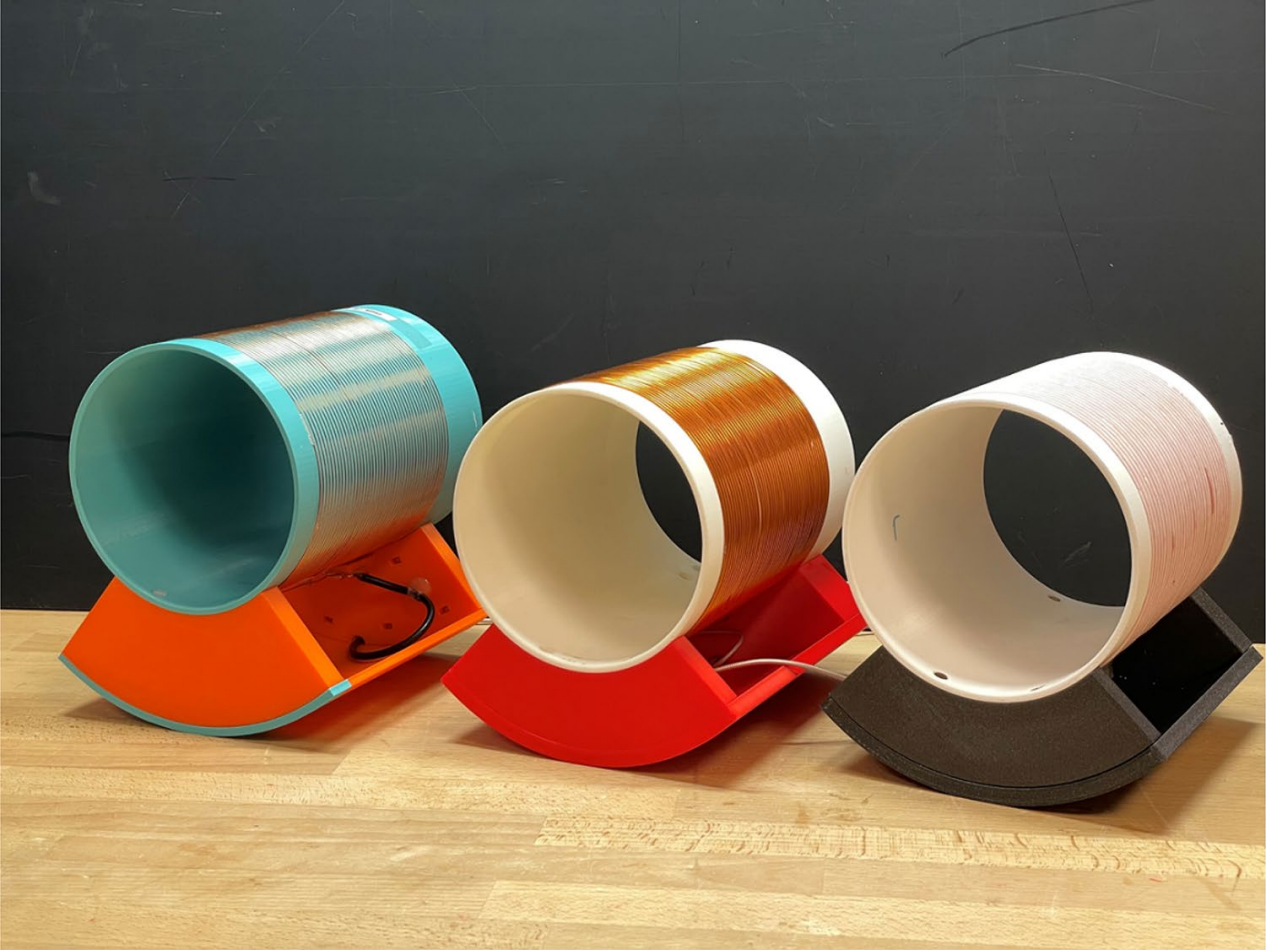
Different solenoidal coils constructed from thin wire (left), thicker wire (centre) and Litz wire (right). The respective unloaded Q values are 88, 240 and 360

Figure 3 exemplifies the principles described in the previous sections. Three solenoidal RF coils were constructed, one with 1 mm diameter copper wire, one with 2 mm diameter copper wire, and one with Litz wire with 1500 strands of 30 μm diameter copper wire. The respective loaded and unloaded *Q* values of the coils were 88, 240 and 360. Each coil is impedance matched to 50 Ω using parallel tuning capacitors ~ 1 nF and series matching capacitors ~ 100 pF.

The SNR is theoretically proportional to the square root of the *Q* value. We performed experiments on a uniform gel-based phantom to test this using the three coils shown in Fig. 3. The respective values were SNR 15.4 (*Q* = 88), 23.5 (*Q* = 240) and *Q* = 31.2 (*Q* = 360).

(c) *Effects of RF shielding*

**Fig. 4:**
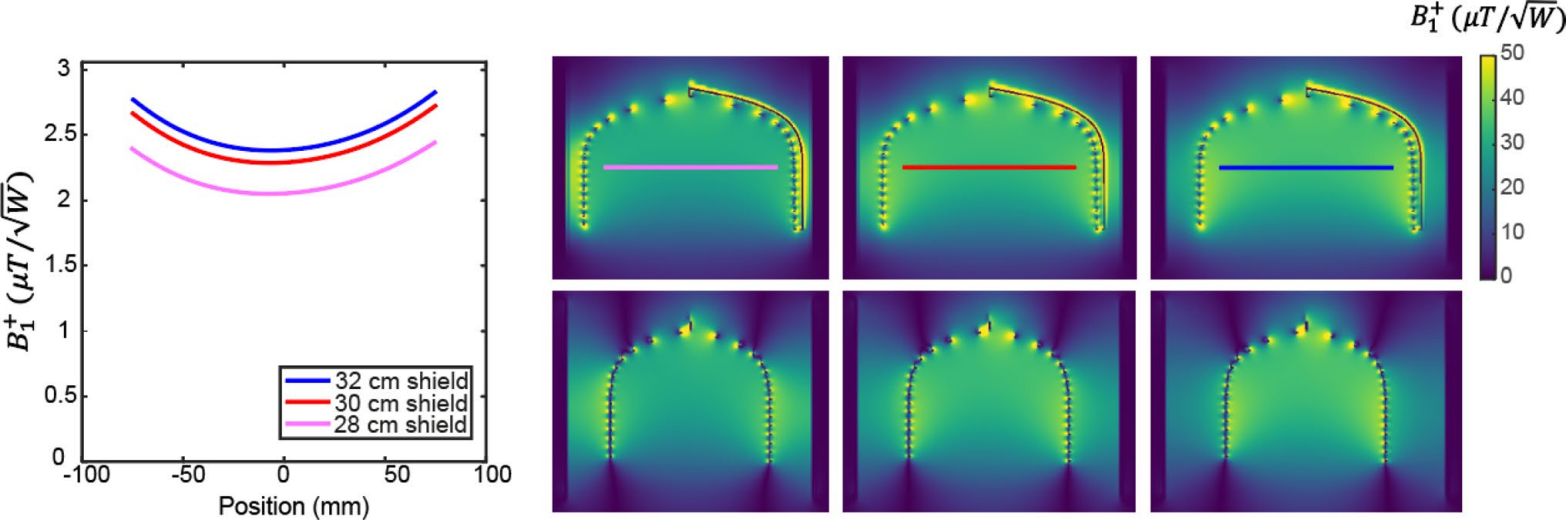
Electromagnetic simulations of the transmit efficiency as a function of RF shield diameter for an elliptical spiral coil with dimensions 180 × 240 × 170 mm. For smaller diameters the field becomes more concentrated between the coil and the shield. The line plots show an ~ 20% increase in transmit efficiency between the 28 and 32 cm diameter shield

As mentioned previously, the compact nature of head scanners means that the conductors of the RF coil are intrinsically very close to the inner Faraday shield. As the shield comes closer to the coil, the transmit field becomes increasingly located between the shield and the RF coil, and the B1 + magnitude in the sample is correspondingly lower. The effect can be analyzed in terms of “mirror currents”, also known as “image currents”, which correspond to counter-currents located outside the shield. The closer the shield to the RF coil, the greater the reduction in transmit efficiency, with a theoretical value of zero if the shield is coincident with the RF coil. This topic is extensively covered in pages 364–371 of Mispelter et al. [74]. An example is shown in Fig. 4, in which electromagnetic simulations were performed using an elliptical spiral coil with dimensions 180 mm width, 240 mm height, and 170 mm length and different circular RF shields with diameters 280 mm, 300 mm and 320 mm. The results show that relative to a 280 mm shield, then there is a 15% increase in transmit (and therefore also receive sensitivity and SNR) if the shield diameter in increased to 300 mm and a ~ 20% increase for a 320 mm shield.

(iii) **Receiver chain**

**Fig. 5:**
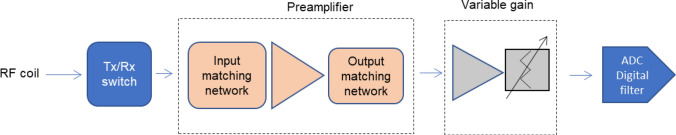
Schematic of the receiver chain. For a receive-only coil the Tx/Rx switch may be omitted if isolation is sufficient

The other major hardware component which affects the overall system sensitivity is the receiver chain, shown in Fig. 5. This consists of a transmit/receive switch (when a single Tx/Rx coil is used), preamplifier, second stage variable amplifier and the analogue-to-digital converter. Each component in the receive chain adds noise, and one can define the reduction in SNR in terms of a noise figure (NF) defined as:

10 $${\text{NF}}\left( {{\text{dB}}} \right) = 20 \log \left( {\frac{{{\text{SNR}}_{{{\text{in}}}} }}{{{\text{SNR}}_{{{\text{out}}}} }}} \right)$$

The overall NF for the entire receive chain can be calculated from:

11 $${\text{NF}}_{{{\text{total}}}} = {\text{NF}}_{{\text{Tx/Rx}}} + {\text{NF}}_{{{\text{preamp}}}} + \frac{{{\text{NF}}_{{{\text{amp}}}} }}{{G_{{{\text{preamp}}}} }} + \frac{{{\text{NF}}_{{{\text{ADC}}}} }}{{G_{{{\text{preamp}}}} G_{{{\text{amp}}}} }}$$

Unlike in much higher frequency MRI systems, the Tx/Rx switch and preamplifier can be placed a significant distance away from the RF coil with negligible cable loss. For example, cable losses in RG 58 at 2 MHz are approximately 1.5 dB per 100 m, with a capacitance of 100 pF per metre.

- *Tx/Rx switch*

**Fig. 6:**
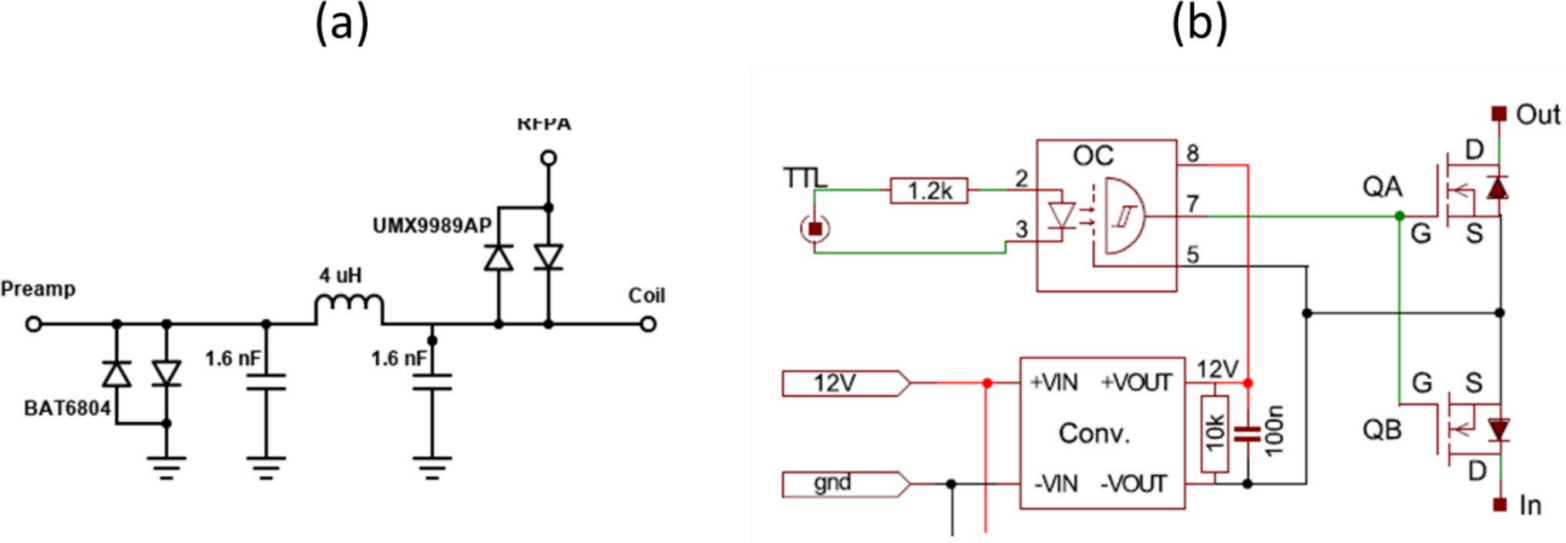
Schematics of **a** a passive Tx/Rx switch designed for operation at ~ 2 MHz and **b** a fast MOSFET switch for operation at frequencies < 150 kHz [76]

Tx/Rx switches can either be passive or active: in either case the most common design is based on the impedance transformation properties of a quarter-wavelength cable, or lumped element equivalent as is much more practical at low-field. Crossed PIN-diode passive designs are essentially the same as those used at higher frequencies, with pi-networks using capacitors and inductors replacing the quarter-wavelength transformer, as shown in Fig. 6a. Active PIN-diode-based switches are also based on this priniciple. PIN-diode drivers and switches for the TRASE experiments at 8 MHz were described by Der et al. [75].

PIN diodes do not work as well at very low frequencies since the RF filters or bias-T schemes used for PIN-diode bias control prevent switching times from being shorter than at least several RF periods. Nacher et al. [76] described the performance of a fast MOSFET-based switch, shown in Fig. 6b, designed for frequencies less than 150 kHz. High power switches using reed relays have been described by Straney et al. again for frequencies in the tens to hundreds of kHz range [26].

As can be seen from equation [11] the NF for the Tx/Rx switch is very important in the overall NF of the receiver chain. A typical loss, which is the same as the NF, for Tx/Rx switches is in the range of 0.2–0.3 dB.

(b) *Preamplifier*

In situations where the receive coil has a loaded *Q* value corresponding to a bandwidth which is much greater than the bandwidth used during image acquisition, then the coil is usually connected to a high gain (20–30 dB), low noise (0.5–1.2 dB) preamplifier with 50 Ω input and output impedance. The rationale at higher frequencies is that the connections between coil and preamplifier are usually via 50 Ω coaxial cable, and therefore to avoid reflections and power loss, all impedances are matched to 50 Ω. At frequencies in the low MHz range, such considerations are much less important: the preamplifier can easily be incorporated into the coil, or even if connected to the preamplifier via a 1 m long coaxial cable, the wavelength is several hundreds of metres and so losses are extremely small and reflections unimportant.

The preamplifier most commonly used in conventional MRI systems is based on a gallium arsenide field effect transistor (GaAs FET), but these are very difficult to obtain commercially at low frequencies, although there are designs in the literature with reported noise figures less than or close to 1 dB [77, 78]. At frequencies in the low MHz range then silicon germanium (SiGe) bipolar transistors are more available commercially, with noise figures ~ 1 dB (e.g., Wenteq Microwave, CA, USA). The input impedance of FETs and bipolar transistors is very high, several tens of kΩ up to MΩ. It is not possible to optimize both NF and gain at the same time. If *Z*_in_ is matched to 50 Ω this gives the maximum gain, but a higher noise figure than desired. For optimal noise matching the condition is that *Z*_s_ = *Z*_opt_, where *Z*_opt_ is the input impedance that minimizes the noise figure of the particular FET.

(c) *Addressing issues associated with very high Q coils*

Having previously stated that the aim of RF coil design is to reduce the coil resistance to its minimum value, one also has to consider that this may result in a very small bandwidth, the effects of which have to be considered with respect both to pulse transmission in terms of the excitation bandwidth of the RF pulses and also signal reception where the receive bandwidth may be greater than that of the RF coil.

As an example of issues in transmission mode, for 3D sequences a pulse width of 100 μs will excite an ~ 10 kHz bandwidth (note that this traditional MR definition is somewhat misleading since the bandwidth refers to the full-width-half-maximum of the approximately sinc-shaped excitation profile, meaning that in practise the tip angle at the maximum offset will have only half its value on resonance), and this means that the linewidth due to *B*_0_ inhomogeneities must be below this value for the entire sample to be uniformly excited. However, for multi-slice excitations the excitation bandwidth of the pulse would be 1.28 kHz for a 3 mm slice thickness using a slice select gradient of 10 mT/m. For a slice 10 cm from the centre of the sample, the offset frequency would be just over 4 kHz. In this case it might be worth to add in an extra resistor during transmission to increase the coil bandwidth such that the tip angle is uniform over all slices. Another reason to reduce the *Q* value in transmit mode is to damp down the coil ringing after the RF pulse. The ring-down can be characterized by an exponential decay with a time constant, *τ*, given by:

12 $$\tau = \frac{2Q}{\omega }$$

**Fig. 7:**
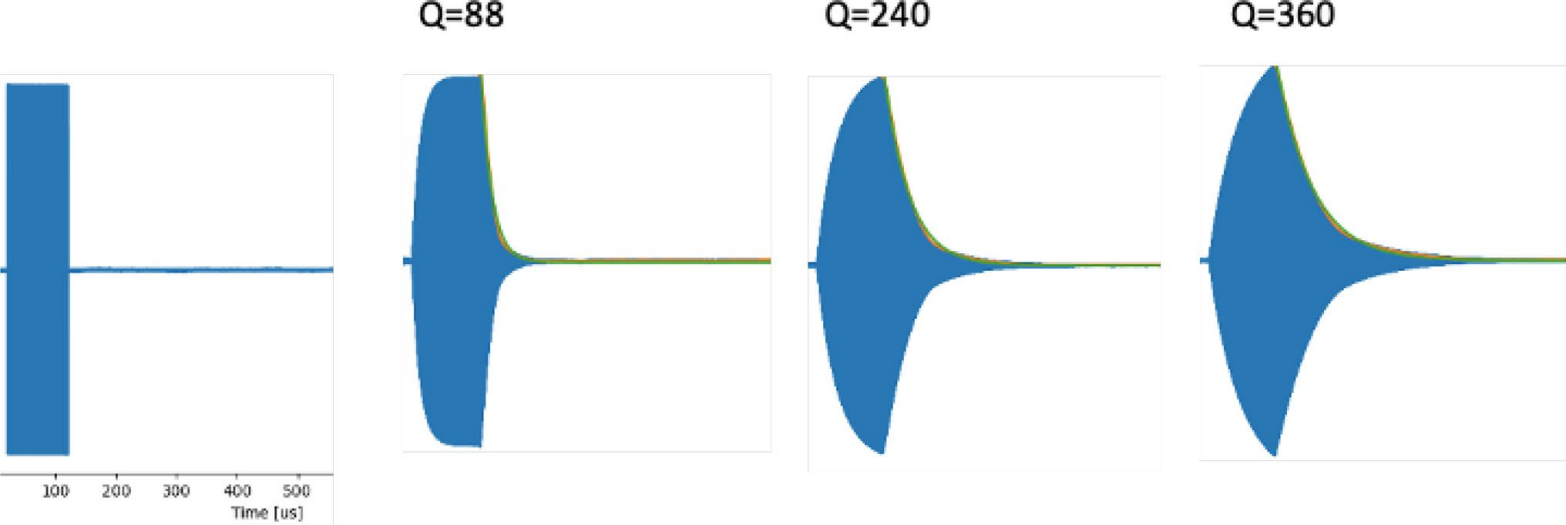
Illustration of the effect of coil *Q* on the measured pulse shape of 100 μs duration hard RF pulse

Many authors have provided solutions to reduce the ring-down time by damping the RF coil at the end of the pulse [78–85], usually by switching in a resistive load or using a modified RF pulse with a negative lobe at the end. Currently most POC systems do not have active Q-damping, according to the descriptions in the literature, since the effects are relatively benign, resulting in a slightly smaller excitation bandwidth than from the ideal pulse shape, with fewer side-lobes. Figure 7 shows pulse ringdown associated with the three different RF coils shown in Fig. 3.

**Fig. 8:**
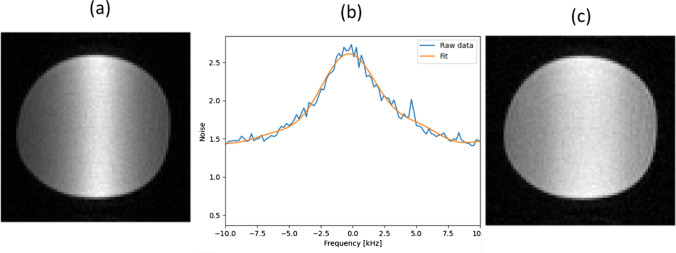
Illustration of correcting the image for the frequency response of a high *Q* RF coil. **a** Image of a uniform phantom using an RF coil with *Q* value of 380. **b** Measured and fitted noise signal. **c** Deconvolution of the image with the fitted polynomial

In receive mode a high *Q* coil can produce banding in the image, as shown in Fig. 8a. There are three well-described ways to deal with the fact that the coil bandwidth may be less, or on the order of, the acquisition bandwidth. One is to “correct” for the artifact by deconvolving by the frequency-dependent coil response function [86]. An equivalent method is to acquire a noise scan, since the noise sensitivity frequency profile reflects the receive sensitivity. A polynomial function is fitted to the noise profile, and the image is then deconvolved using this function, as shown in Fig. 8b and c, respectively.

**Fig. 9:**
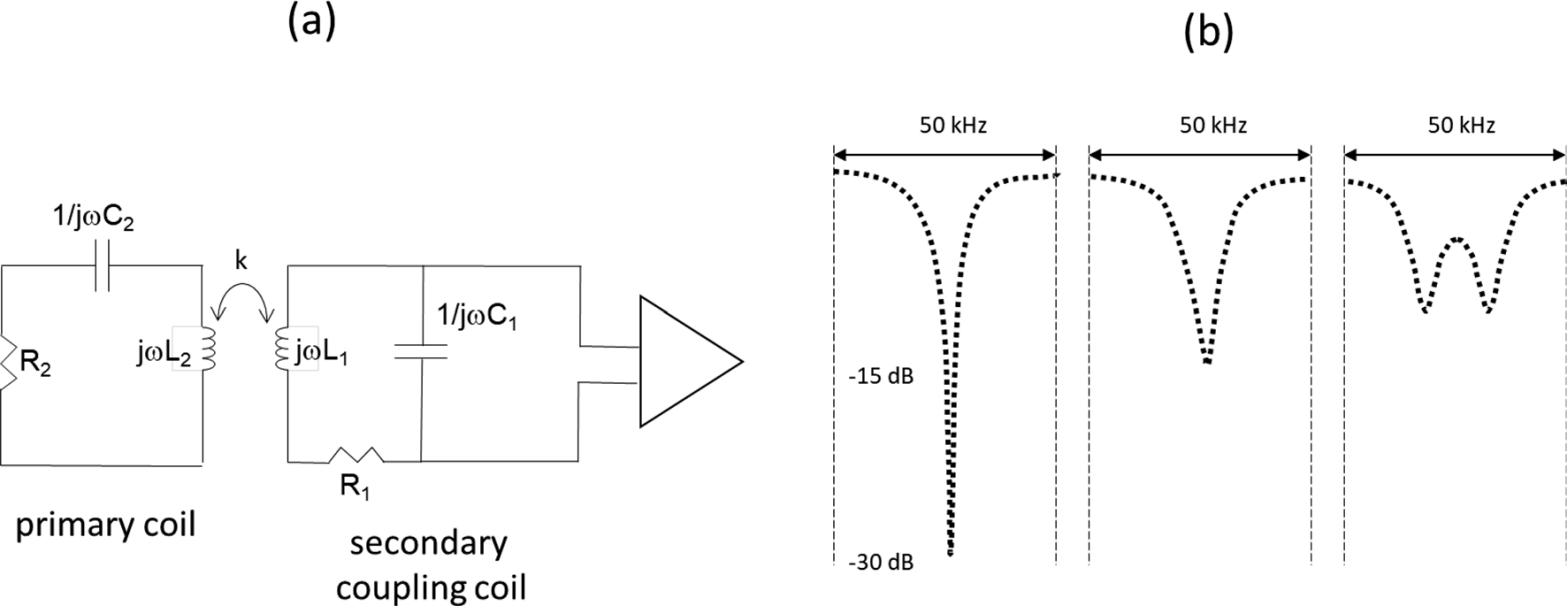
**a** Increase in the effective receive bandwidth using strong coupling between a secondary coil and the primary receive coil. The effects of strong overcoupling on receive bandwidth and sensitivity. **b** Measured *S*_11_ parameters corresponding to, from left-to-right, weak, critical and overcoupling

The second method is to use inductive coupling between a secondary coil and the primary receive coil, as shown in Fig. 9a: the output of the coupling coil is connected directly to a high input impedance preamplifier. Raad and Darrasse showed at 0.1 T [87] that this increased the bandwidth by a factor-of-five with an SNR reduction of less than 1 dB. To significantly increase the bandwidth of the preamplifier, the coupling must be strong (overcoupled), with ideally the *Q* value of the secondary coil being much larger than that of the primary coil. Under these conditions, the 1 dB bandwidth is given by:

13 $$B_{{{\text{1dB}}}} = 0.46\omega_{{\text{p}}} k\sqrt {\frac{{Q_{{\text{s}}} }}{{Q_{{\text{p}}} }}}$$

where the subscripts s and p refer to secondary and primary, respectively, and k is the coupling constant. Figure 9b shows the corresponding S-parameter plots.

The third method is to use a strong impedance mismatch between the RF coil and the preamplifier to effectively broaden the response. In this case, a preamplifier that is noise-matched rather than power-matched reduces the effective coil *Q*-factor without reducing the SNR [78, 79, 88–90] as shown in the analysis below.

**Fig. 10:**
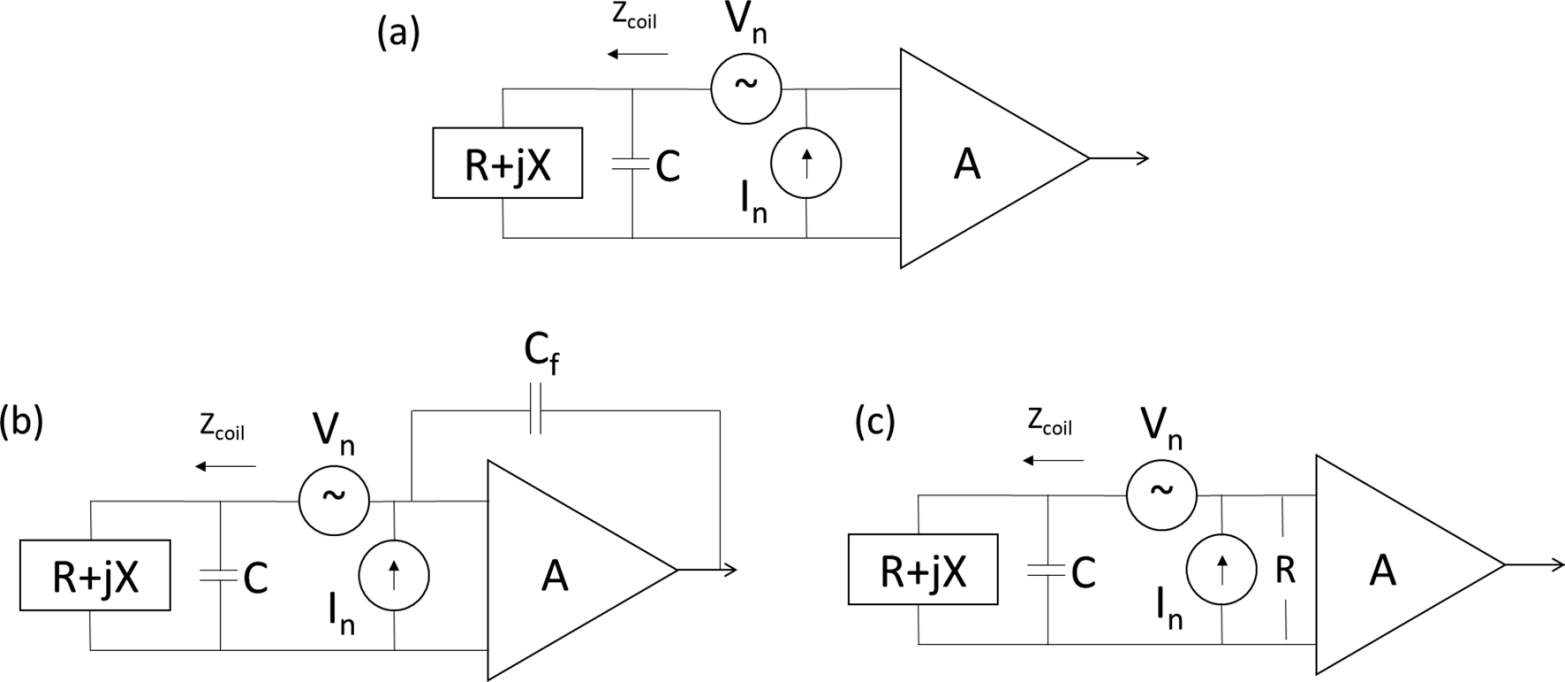
**a** simplified model of an RF coil with resistance and inductive reactance, tuned with capacitor *C*, connected to a preamplifier with gain *A*, and voltage and current noise sources *V*_n_ and *I*_n_, respectively. **b** Capacitive feedback can be used to increase the effective bandwidth of the preamplifier. **c** An equivalent circuit which has a noiseless resistor across the input of the preamplifier, therefore reducing the *Q* of the receiver coil without introducing extra noise

A model of a preamplifier, shown in Fig. 10a, contains independent voltage and current noise sources, *V*_n_ and *I*_n_, respectively. Assuming that the current and voltage noise sources in the amplifier (and all other noise sources in the circuit) are uncorrelated, then the NF is given by:

14 $${\text{NF}} = 1 + \frac{{I_{n}^{2} \left| {Z_{{{\text{coil}}}} } \right|^{2} + V_{n}^{2} }}{{4kTR_{{{\text{coil}}}} }}$$

which importantly shows that the NF is *independen*t of the impedance across the inputs of the preamplifier. The highest SNR occurs when the RF coil and the preamplifier are noise matched as opposed to power matched. The noise matching condition is given by a purely resistive impedance:

15 $$R_{{{\text{opt}}}} = \frac{{V_{n} }}{{I_{n} }}$$

JFET transistors have optimum values ranging from 10 kΩ to 1 MΩ. A typical RF coil at low frequencies has a low resistance and is inductive (positive reactive impedance), and therefore a tuning capacitor is needed to resonate the coil, in other words to give the maximum impedance. At resonance the coil impedance is given by:

16 $$R_{{{\text{coil}}}} = \omega LQ$$

One way of increasing the effective receiver bandwidth is by incorporating a feedback loop into the preamplifier circuit, as shown in Fig. 10b, which is equivalent to adding a noiseless resistor in parallel to the input of the preamplifier, Fig. 10c, thereby changing its input impedance. The increased resistance reduces the *Q* of the detection circuit, thus increasing the effective bandwidth. The concept of using negative feedback in the receiver [78, 88–90] has been extensively analyzed in particular in the papers of Hoult and Kuzmin. Optimal low noise preamplifier and matching design has also been considered in the area of magnetic particle imaging [91].

The effect of the feedback capacitance on the receiver is an integrating transfer function, *H*(*ω*), given by:

17 $$H\left( \omega \right) = \frac{j}{\omega \tau }$$

where *τ* is the integrating time constant. The effective input admittance is given by:

18 $$Y_{{{\text{in}}}} = j\omega C_{{\text{f}}} + \frac{{C_{{\text{f}}} }}{\tau }$$

Provided that *τ* >  > 1/*ω*, this is equivalent to a noiseless resistor with value *τ*/*C*_f_ in parallel with the input to the preamplifier.

(d) **Data acquisition system**

In line with the concepts of portability, sustainability and accessibility, most of the data acquisition systems used for POC MRI are much simpler and more compact than those associated with clinical MRI systems. This also implies that many of the features that are built into clinical systems are not present and must be added if required. Examples include eddy current compensation, gradient trimming, automatic RF power calibration, and image-based shimming. Most consoles used for POC MRI allow pulse sequence programming using relatively simple software. The key elements of a console are the timing accuracy and reproducibility of sending out both RF (magnitude and phase) and gradient pulses, accurate locking of data acquisition to RF and gradient signal transmission, the dynamic range and number of bits of the receiver, and the characteristics of the digital filters used for oversampling the data.

Examples of commercial spectrometers used for POC data acquisition include the Magritek Kea 2, the MR Solutions EVO 2, Pure Devices, Tecmag Bluestone, and RS2D Cameleon 4. Taking the Kea 2 as an example, the specifications of the system include direct digital synthesis used on the transmit side, signal reception at a fixed 100 MHz oversampled frequency, a 16-bit ADC. An inbuilt 50 Ω nominal input impedance preamplifier has a gain of 37 dB and noise figure < 1.5 dB: a passive duplexer is used as a transmit/receive switch. The Apollo Redstone has the following specifications: 10 ns minimum pulse width, 10 ns timing resolution, < 10 ns phase switching 0.0055° phase resolution, < 20 ns phase-continuous frequency switching, 64 million point waveform memory for each transmitter and gradient channel, 12.5 MHz (80 ns per complex point) digital receiver bandwidth, and up to 24-bits of digital receiver dynamic range.

Even though these consoles are significantly less expensive than their clinical counterparts, they still represent a significant fraction of the total cost of the POC system, and may also be based on proprietary software and hardware. As a result, there have been a number of different open-source MRI consoles described in the literature [92–94], taking advantage of the rapid advances in digital technology and open-source software. In designing such systems, it is important to note that almost all of the hardware elements were originally produced for purposes other than MRI, and so it is key that features such as phase stability, inter-shot reproducibility, etc., which are very important for optimal MRI performance, are thoroughly investigated for particular designs. Michal has published a recent review of hardware which is relevant to the 10 s to 100 s kHz range [94]. Among the projects opened to the community [92–95] the Open-source Console for Real-time Acquistion (OCRA [95]) is notable for its flexibility, inexpensive off-the-shelf components, community focus, and real-time capabilities. The MAgnetic Resonance COntrol System (MaRCoS) [96] uses the same versatile hardware, however its software, firmware and FPGA firmware have been replaced to go beyond the limitations of OCRA, which included limitations on sequence length, complexity and timing precision,as well as the being written in low-level ‘assembly’-style sequence programming. MaRCoS represents a complete rewrite of the server, FPGA firmware, and client software, based on a msgpack-based protocol which also supports the open-source PulSeq hardware-independent sequencing language [97]. The core of MaRCoS is the Red Pitaya SDRLab 122–16, a commercial board with two analog inputs for digitizing received data and two analog outputs for generating the RF transmit waveforms, with a bandwidth of around 50 MHz making it suitable for proton MRI at field strengths of up to 1.17 Tesla. Two receive/transmit channels are run in parallel, with frequency up/down-conversion and the bulk of the filtering handled digitally. Three digital outputs can be used for controlling RF switches or other peripherals, and one input is used for externally triggering acquisitions. The SDRLab also controls either a GPAFHDO or an OCRA1 four-channel gradient board. The MaRCoS hardware is controlled from a PC over Ethernet. On the SDRLab, sequence and acquisition data are streamed to and from an FPGA, allowing for sequences of arbitrary length. Sequences are programmed at a low level using simple arrays of times and values for each parameter, which are converted to hardware instructions by the marcos client Python library. There are several intermediate text-based interfaces to suit different styles of programming, as well as a GUI for running a range of calibrations and predefined acquisition routines.

(v) **Grounding and shielding**

Two other practical issues in terms of maximizing SNR are proper grounding of all the system components and RF shielding. There are multiple active (gradient amplifiers, RF amplifier, data acquisition system) and passive (external RF shield, internal RF shield, patient table) components which represent different grounding points. With proper grounding and filtering, connecting the RF and gradient amplifiers should ideally not add measurable noise [68]. However, it is well-known that the human body acts as an antenna for any external EMI and “guides” this EMI into the imaging region [98]. A conductive mesh cloth can be draped over the patient to reduce the body antenna effect [29, 99], but this set-up can be somewhat uncomfortable and relies on very good contact with any local shielding structures [33].

**Fig. 11:**
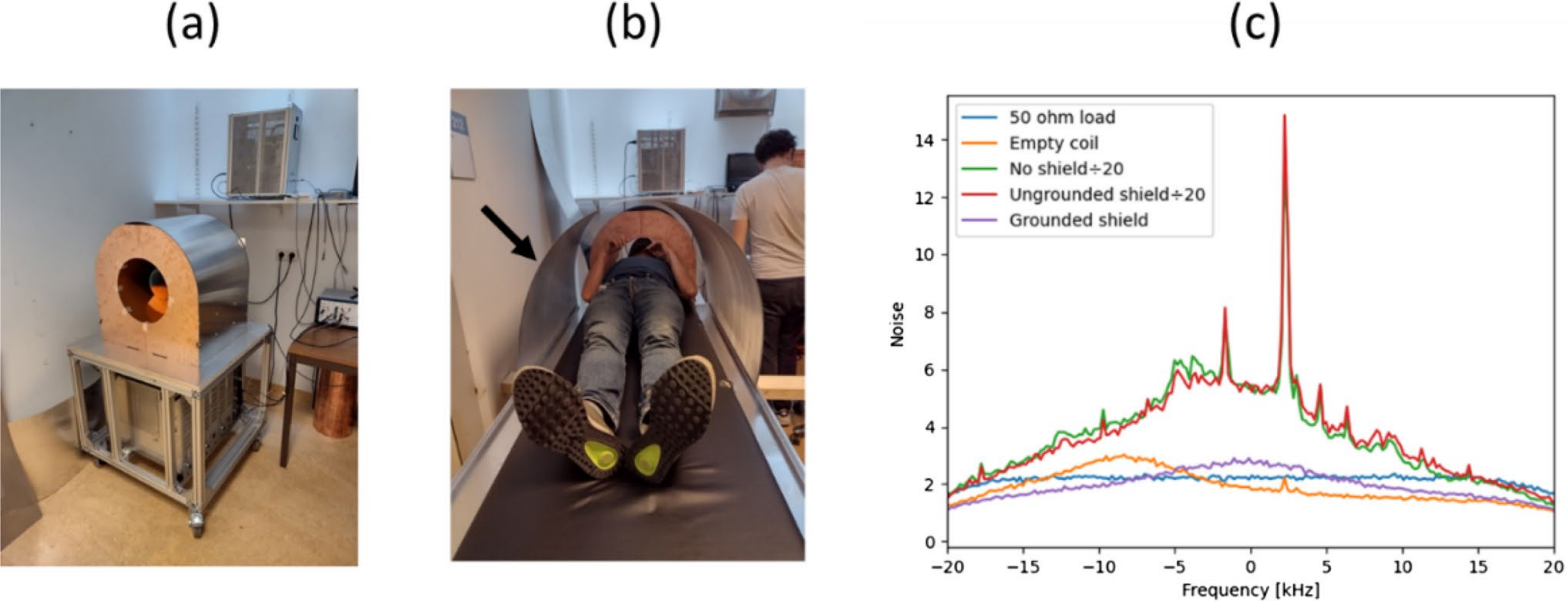
**a** Photograph of the low-field POC system at Leiden, showing shielded magnet and grounding plate on the portable trolley. **b** Photograph of the thin semi-cylindrical aluminium RF shield which is placed around the subject being imaged. **c** Illustration of the noise levels associated with different operating and grounding conditions

Figure 11 shows the very different noise levels that can result from different shielding and grounding conditions. In this case the system is not in a shielded room (which can reduce external sources of broadband noise by > 100 dB). The worst case occurs when the human subject is placed in the RF coil, and there is no RF shield in place, or even if the shield is in place, it is not grounded to the rest of the system. If the shield is grounded then the noise level drops by approximately a factor of 100 to a level which is almost identical to that of an unloaded coil. Similar reduction levels have been reported using conductive cloth, although authors also report that this approach is very much subject-specific [28] and the cloth typically loses performance over time.

(f) **Active cancellation of external electromagnetic interference**

Ideally, POC scanners are designed to work in challenging environments such as the ICU, emergency room, and in remote locations. Even with the system fully grounded and shielded, as described in the previous section, strong sources of electromagnetic interference (EMI) from nearby medical equipment, or general environmental EM noise, can cause artifacts which render images non-diagnostic. Many approaches for EMI reduction in MRI/NMR have been explored in the past [100–106], with most approaches consisting of data post-processing using signals picked up from external sensors, such as additional RF coils. This type of approach has been implemented on the Hyperfine Swoop system, and was also recently shown by Srinavas et al. [33] on their 80 mT low field POC system which has multiple receive channels. A machine-learning approach for EMI cancellation has been presented by Liu et al. [34] using ten EMI-sensing coils placed close to the transmit and receive coils, underneath the patient bed, and inside the electronics cabinets. EMI interference detected by both the sensing coils and MR receive coils were used to train a five-layer convolutional neural network (CNN) model. This model was used to predict the EMI signal component in the MRI receive coil based on the signals picked up by the sensing coils: this EMI component was then subtracted from the image.

### A summary of POC system characteristics from the literature

The hardware designs for a few different POC systems have been described in the literature. This section summarizes the relevant characteristics of the systems in terms of those features which directly affect the SNR.

Cooley et al. [28], operating at 80 mT (3.39 MHz) designed a Tx/Rx spiral helmet, with inner dimensions 21 cm (anterior–posterior), 17 cm (medial–lateral), matched to 50 Ω. Twelve asymmetric windings of Litz wire (AWG 20 5/39/42), with a higher turn-density near the bottom of the coil were used to optimize B_1_ homogeneity: the windings extended 10.7 cm from the top of the head and the coil had an inductance of 69 μH A 50 Ω input impedance, 37 dB gain pre-amplifier (MITEQ model AU-1583, Hauppauge, NY, USA) and a 24 dB second stage amplifier (Minicircuits model ZFL-500LN + , Brooklyn, NY, USA) were used. A similar geometry coil was used in work [33] at 47 mT. In this latter case a 50 Ω, 70 dB gain pre-amplifier (MITEQ P/N 1583 10 057, NY, USA) was used.

(i) 80 mT magnet with a built-in gradient of 7.6 mT/m,
(ii) loaded and unloaded Q values were 150 and 225, respectively
(iii) a hard 90° pulse of 80 μs duration required an input power of 44 W, corresponding to a transmit efficiency of 11 μT/√W.

He et al. [35], operating at 50.9 mT, used separate transmit and receive coils. The transmit coil was a variable-density elliptical solenoid of 8 turns of copper wire, three either end and one in the centre with length 24 cm and 29.4 × 24.8 cm diameter in the long- and short-elliptical axes. A PIN diode was used to detune the transmit coil during signal reception. The receive coil was a solenoid coil with elliptical cross-section (major axis diameter 23 cm, minor axis diameter 19 cm) constructed from enameled copper wire (2-mm diameter). An active and passive detuning module was added in the matching circuit to detune the receiver coil during RF transmission.

(i) the receive coil had *Q* values of 38.7 unloaded and 37.4 loaded.

Liu et al. [34], operating at 55 mT also used separate transmit and receive coils. The receive coil was a solenoid coil with an elliptical cross-section (vertical axis 23 cm and horizontal axis 19 cm) with 10 turns and 9.5 cm length. A decoupling circuit was also implemented to detune the receive coil during RF transmission. Liu used a two-stage preamplifier module (first-stage: Gain = 30 dB; second stage: Gain = 30 dB, for input Vpp < 60 mV).

(i) The transmit solenoid used 11 W input power for a 1 ms 180° pulse, corresponding to a transmit efficiency of 3.6 μT/√W
(ii) The *Q* factors of the receive coil were approximately 30 loaded and 31 unloaded.

Guallart-Naval et al. [99] designed a 70 mT Halbach array for musculoskeletal applications.

The transmit/receive coil was a standard solenoid. The receiver chain used a low-noise preamplifier with ~ 45 dB gain.

(i) The transmit/receive coil had loaded/unloaded *Q* values of 88/93.

**Fig. 12:**
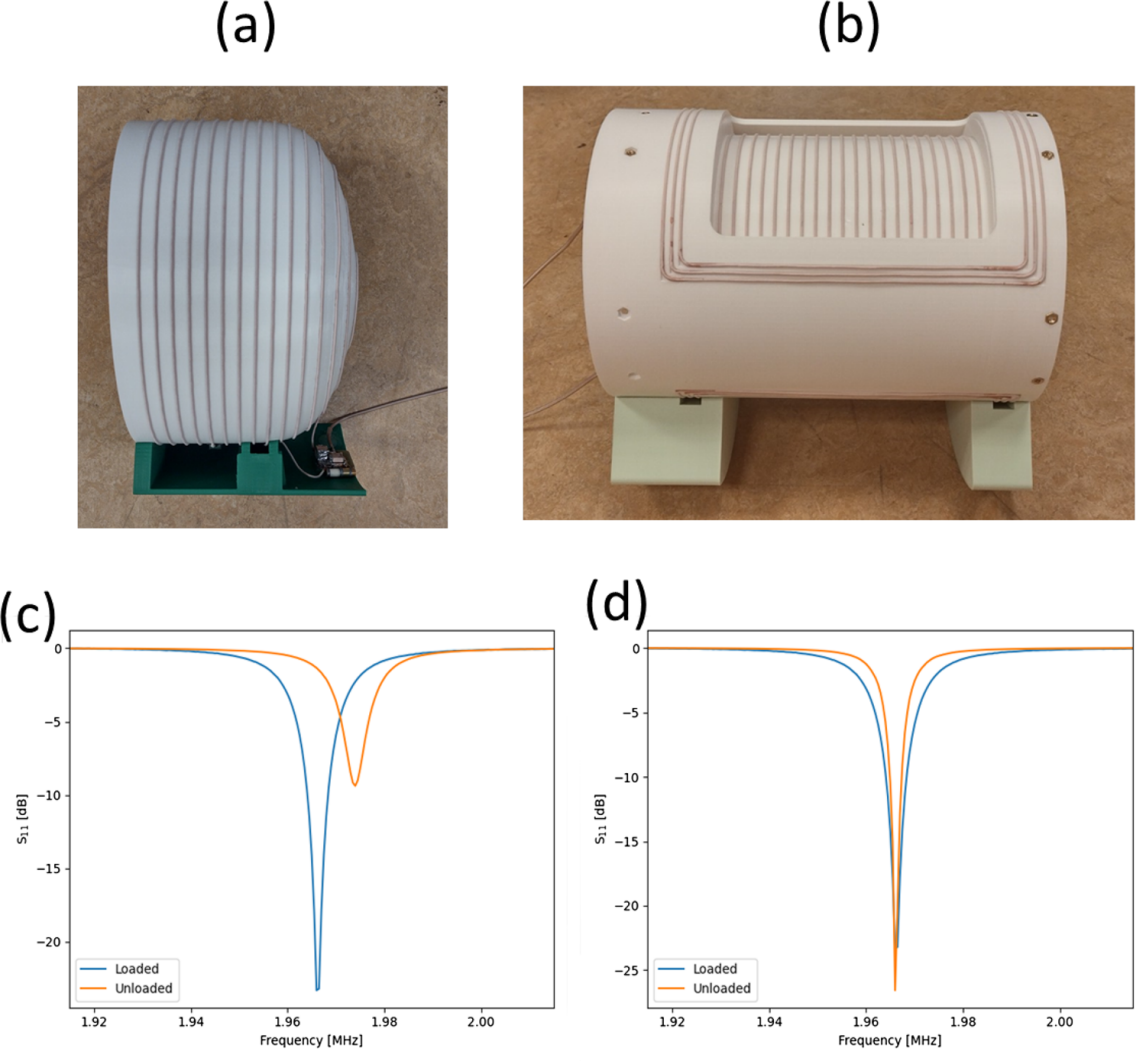
Photographs of **a** the spiral Litz wire coil used for neuroimaging at 47 mT, and **b** the separate Tx (saddle) and Rx (solenoid) Litz coils used for knee imaging. **c** Plot of *S*_11_ with the blue line representing the impedance matched RF coil loaded with the head, and orange line the unloaded case without re-tuning. **d** Corresponding plot with the orange line now obtained after impedance matching for the unloaded case

Figure 12 shows the RF coils used for neuroimaging and knee imaging in Leiden operating at 47 mT. The spiral solenoid has dimensions 235 × 180 × 140 mm, 15 turns, Litz wire (1500 × 30 μm diameter). The knee saddle transmit coil has dimensions 185 × 185 × 220 mm, 3 turns, Litz wire (1500 × 30 μm diameter), the knee solenoid receive coil has dimensions 155 × 155 × 150 mm, 20 turns, Litz wire (1500 × 30 μm diameter). The network analyzer measurements show the change in S_11_ between the unloaded and loaded cases for the spiral head coil, which shows that there is a significant difference both in resonant frequency and impedance matching between the loaded and unloaded cases which must be considered.

(i) The spiral transmit/receive coil had loaded/unloaded *Q* values of 215/350.
(ii) The spiral transmit/receive solenoid used 2.5 W input power for a 100 μs 90° pulse, corresponding to a transmit efficiency of 37 μT/√W.
(iii) The knee saddle transmit coil had loaded/unloaded *Q* values of 190/243
(iv) The knee solenoid receive coil had loaded/unloaded *Q* values of 240/400

**Fig. 13:**
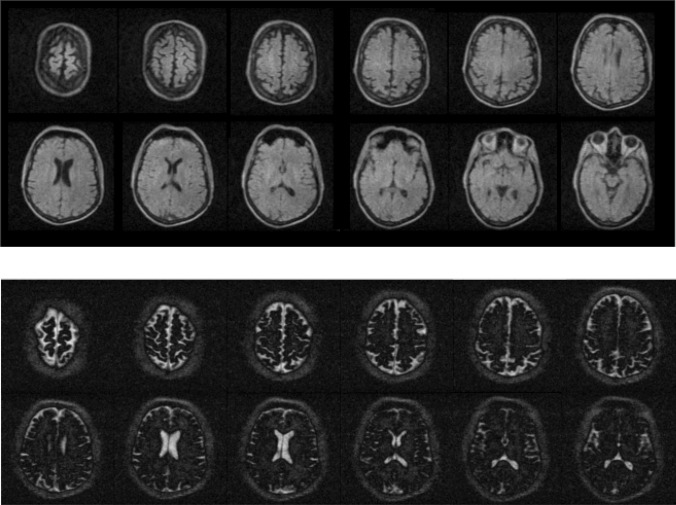
In vivo images acquired at 47 mT with the high *Q* coil. (top) TSE sequence: field-of-view 220 × 220 × 225 mm, acquisition matrix 110 × 110 × 75, TR/TE 600/15 ms, echo train length 7, elliptical *k*-space coverage, no signal averaging, acquisition bandwidth 20 kHz, data acquisition time 9 min. (bottom) Inversion-recovery TSE: field-of-view 220 × 220 × 180 mm, acquisition matrix 110 × 110 × 60, TR/TI/TE 6000/200/18 ms, echo train length 110, elliptical *k*-space coverage, no signal averaging, acquisition bandwidth20 kHz, data acquisition time 6.2 min

Images of the brain of a healthy volunteer acquired using the Kea-2 spectrometer with a turbo spin echo sequence are shown in Fig. 13.

## Discussion

There are many different POC systems that are being used for neuroimaging and musculoskeletal applications. Current POC systems have the following general characteristics in terms of the magnet and RF coil designs:

(i) *B*_0_ field strengths between ~ 45 and ~ 80 mT
(ii) Either Tx/Rx or separate Tx and Rx RF coils, constructed either from Litz wire (unloaded *Q* values ~ 300–400) or copper wire (diameter ~ 2 mm, unloaded *Q* values ~ 30—here one should note that with larger diameter copper wire much higher unloaded *Q* values should be reachable). In Tx mode it is important to consider that too high a *Q* value would result in a transmit bandwidth which might cause tip angle and phase variations across the sample if the *B*_0_ homogeneity is very poor, or if two-dimensional multi-slice experiments are performed,
(iii) *Q*-damping to reduce coil ringing in transmit mode is generally not used,
(iv) 50 Ω input impedance preamplifiers are predominantly used, accompanied by various image processing methods to reduce the effect of the limited receive bandwidth of high *Q*-coils (if present).

With many different types of POC low field MRI systems being developed, it is challenging to assess the performance of each different approach, especially as each element of the system (magnet, gradient coils, RF coil, gradient amplifier, RF amplifier, preamplifier and data acquisition system) may be unique to a particular configuration. One approach to performance quantitation is to design phantoms for low-field MRI, analogous to that of the American College of Radiology (ACR) for clinical field strengths, which can provide information on parameters such as SNR, geometric accuracy, high contrast spatial resolution, slice thickness accuracy, slice position accuracy, image intensity uniformity, percent signal ghosting, and low-contrast object detectability. A second approach which we are currently pursuing is to determine how close we are to the ultimate SNR, an approach which has been investigated extensively at higher fields [107, 108].

As a community, it would be very helpful to give as much information as possible on the characteristics and performance of individual elements of the POC system. With respect to the sensitivity, this includes the unloaded and loaded *Q* values of the transmit and receive coils, the transmit and receive sensitivities, preamplifier noise figure and gain, and specific details of the acquisition bandwidth, *k*-space coverage and the particular form of *k*-space or image domain filter. A standardized SNR mapping protocol and image reconstruction/processing pipeline could be helpful in achieving this goal. In addition to the information and parameters listed, investigators should report the details of their image reconstruction (especially the coil combination method if multi-channel Rx arrays are used, i.e., sum-of-squares vs. noise-covariance weighted optimal coil combination).

## Data Availability

Data are available upon request from the authors.
